# From adoption to interaction: examining human-GenAI engagement patterns, ethical tensions, and educational trade-offs in nursing education

**DOI:** 10.1186/s12912-026-05132-7

**Published:** 2026-07-31

**Authors:** Meichun Yang, Jiahui Long, Yaolei He, Ruifeng Meng, Caixia Ling, Qijun Long, Jianjun Wen

**Affiliations:** 1 Affiliated Hospital of Youjiang Medical University for Nationalities, Baise, Guangxi 533000 China; 2 School of Nursing, Philippine Women’s University, Manila1004, Philippines; 3 Medical College, Xinjiang University of Science & Technology, Korla, Xinjiang 841009 China; 4 College of Humanities and Management, Youjiang Medical University for Nationalities, Baise, Guangxi 533000 China; 5 Center of International Cooperation and Exchange, Youjiang Medical University for Nationalities, Baise, Guangxi 533000 China

**Keywords:** Human–GenAI interaction, Generative AI in higher education, Nursing education, Student engagement, DeepSeek

## Abstract

While generative AI (GenAI) tools such as DeepSeek are increasingly embedded in higher education, existing research has predominantly focused on accuracy, bias, and adoption, with limited attention to the nature of human–GenAI interaction in learning. This study examines how nursing students interact with GenAI and how distinct interaction patterns shape cognitive, behavioral, and affective outcomes, as well as ethical risks in professional training contexts. A cross-sectional mixed-methods design was employed, integrating a rapid review, survey data (*n* = 401), and qualitative interviews (*n* = 30). Quantitative data were analyzed using descriptive statistics, correlations, and regression, while qualitative data underwent thematic analysis to identify recurring interaction patterns. Four dominant interaction patterns emerged: (1) passive consumption, characterized by uncritical acceptance of outputs; (2) efficiency-driven use, where GenAI functions as a productivity shortcut; (3) dependent interaction, reflecting overreliance and reduced self-directed learning; and (4) critical engagement, involving verification and reflective use. These patterns are associated with divergent outcomes. Efficiency-driven and dependent interactions were associated with greater perceived task efficiency and performance, but were also associated with lower critical thinking, reduced motivation, and increased academic dishonesty. In contrast, critical engagement supports deeper learning but demands higher AI literacy and effort. Ethical risks—including misinformation, fabricated references, and misalignment with clinical standards—are most pronounced under passive and dependent use. The findings suggest that educational outcomes are shaped not merely by GenAI adoption but by interaction quality. This study advances a shift toward interaction-centered frameworks, emphasizing the need for structured, critical, and ethically grounded GenAI engagement in higher education.

**Supplementary Information:**

The online version contains supplementary material available at https://doi.org/10.1186/s12912-026-05132-7.

## Introduction

Artificial intelligence (AI) refers to computational systems capable of performing tasks commonly associated with human intelligence, including prediction, language processing, problem-solving, and decision support. *Generative artificial intelligence (GenAI)* is a subset of AI that produces new content—such as text, explanations, summaries, and responses—based on patterns learned from large datasets. In this study, *DeepSeek* refers to a large language model that generates context-sensitive textual responses to users’ prompts and can be applied to academic and clinical learning tasks. AI and GenAI are rapidly transforming healthcare, education, and public policy by offering efficiency, personalization, and predictive support [7, 11, 43]. Recent international surveys indicate that more than 80% of university students have used generative AI tools for academic purposes, while healthcare and nursing students increasingly utilize large language models for literature searches, concept clarification, assignment preparation, clinical reasoning exercises, and examination review [20, 23, 28]. Similarly, the global artificial intelligence market in healthcare is projected to exceed USD 180 billion by 2030, reflecting the accelerating integration of AI into healthcare education and professional practice [43].

Current discourse has largely emphasized concerns related to accuracy, bias, plagiarism, academic integrity, and professional accountability [16, 32, 34, 53]. While these issues remain central, they do not fully explain how educational outcomes are shaped through the *interaction processes* between learners and generative AI. Scholars caution that generative AI’s potential to foster misinformation, over-reliance, and reduced critical judgment could undermine the ethical foundations of nursing education if left unchecked [12]. These risks are not solely a function of the technology itself, but of how students engage with, interpret, and act upon AI-generated outputs. Such concerns mirror broader debates in healthcare, where transparency, trust, and explainability remain unresolved challenges [24].

China presents a particularly relevant context for examining these dynamics, given its aggressive national innovation strategies driving AI integration in healthcare and higher education [40]. As one of the world’s leading investors in artificial intelligence, China has incorporated AI development into its national education and healthcare modernization strategies, encouraging universities to integrate intelligent technologies into teaching, clinical simulation, and research. Nursing schools have increasingly adopted generative AI applications to support personalized learning, virtual tutoring, documentation training, and clinical decision-support exercises, creating new opportunities as well as emerging ethical and pedagogical challenges [39, 40, 44]. Within nursing education, AI tools are increasingly embedded in instruction, simulation, and assessment. However, the absence of standardized ethical and pedagogical frameworks means that students must navigate these technologies through their own interaction practices, often without guidance [29, 39, 42]. In high-stakes domains such as clinical reasoning and decision-making, the quality of human–AI interaction becomes critical, as uncritical acceptance, overreliance, or superficial engagement may compromise both learning outcomes and professional competence. Cultural norms, hierarchical academic traditions, and evolving regulatory policies further shape how students interact with AI, complicating the ethical and pedagogical landscape. Despite China’s leadership in AI innovation, vulnerabilities in *interaction practices* within nursing education remain insufficiently understood [40, 44].

Recent advances in large language models (LLMs) have substantially expanded their capabilities in medical and health professions education. The emergence of large biomedical knowledge bases, coupled with the capabilities of language models in understanding natural language and supporting clinical decision-making, has led to the identification of foundation models as a new class of generalist medical AI systems that can bring together biomedical knowledge, clinical reasoning, language understanding, and decision support across a wide range of medical specialties [36]. In a short span, LLMs have advanced from basic conversational tools to complex educational aids that can provide clinical explanations, summarize scientific research, facilitate evidence-based learning and conduct simulated clinical reasoning exercises [8]. Furthermore, these abilities were further boosted by the use of reinforcement learning techniques, such as DeepSeek-R1, which was developed to improve the quality of reasoning and problem-solving performance by leveraging reinforcement learning instead of only conventional supervised fine-tuning [25]. The advancements of these technologies indicate that current LLMs have come to play a more important role in the educational context, especially in areas like nursing education, where they can assist with analytical thinking, evidence-based decision making, and ongoing learning.

More recent comparative tests also illustrate the high performance state of the art LLMs can obtain on specialized medical evaluations, sometimes reaching or even surpassing human standards in some areas. ChatGPT-4.0, Gemini Advanced, and DeepSeek, for instance, have shown promising results on ophthalmology knowledge tests and clinical reasoning exercises, with certain models outperforming others in terms of reasoning accuracy, factual correctness, and consistency [22, 31]. Nevertheless, concerns remain regarding hallucinated information, fabricated references, misinformation, and the rapid dissemination of inaccurate health information, giving rise to what has been described as an AI-driven “infodemic” in healthcare and public health [14]. Thus, it is not possible to accurately assess the educational value of GenAI based on benchmark performance or response accuracy. Instead, it will rely on learners’ critical thinking, verification and integration of AI-generated information in their clinical and academic reasoning. Although LLM capabilities are rapidly growing, there remains a lack of empirical studies analyzing these interaction processes between nursing students, which leaves many questions about effective, ethical, and educationally significant human–GenAI interactions unanswered.

The emergence of DeepSeek further accentuates these gaps. As a large language model tailored for Chinese contexts, DeepSeek offers culturally relevant, multilingual, and discipline-specific capabilities. Since its public release, DeepSeek has attracted widespread adoption across Chinese universities because of its strong Chinese-language reasoning capability, open-source architecture, and competitive performance compared with other large language models in academic and healthcare tasks [2, 50, 51]. However, empirical research has primarily examined its technical performance, with limited attention to how students *interact* with the system in real learning contexts [40, 50–52]. Persistent concerns regarding bias, misinformation, dishonesty, and overreliance highlight the need to move beyond evaluating AI outputs toward understanding interaction dynamics [1, 4]. For example, a nursing student may copy a medication explanation generated by DeepSeek directly into an assignment without verifying its clinical accuracy, representing passive interaction. Another student may use DeepSeek primarily to summarize lengthy lecture notes or generate practice questions before examinations, reflecting efficiency-driven interaction. In contrast, a student who cross-checks AI-generated care plans against nursing textbooks, clinical practice guidelines, or instructor feedback demonstrates critical engagement. Likewise, repeatedly relying on AI to answer dosage calculations, develop nursing care plans, or formulate clinical rationales without independent reasoning exemplifies dependency-based interaction. These examples illustrate that the educational consequences of GenAI depend less on whether students use AI and more on how they interact with it during learning. In particular, there is a lack of empirical evidence on how these distinct interaction patterns shape cognitive, behavioral, and affective outcomes in nursing education.

Addressing this gap, the present study shifts the analytical focus from AI adoption to *human–GenAI interaction*. Using a cross-sectional mixed-methods design, the study examines how nursing students engage with DeepSeek, identifying distinct interaction patterns and analyzing how these interactions influence educational outcomes and ethical tensions. Quantitative data capture relationships among awareness, interaction tendencies, and usage intention, while qualitative findings provide insight into students’ lived experiences, interpretive processes, and decision-making behaviors when using AI. By triangulating these strands, the study offers an empirically grounded account of how interaction practices mediate both the benefits and risks of generative AI in nursing education. In doing so, it contributes to a more nuanced understanding of effective and ineffective human–GenAI interaction and provides a foundation for developing evidence-based approaches to integrating AI in higher education.

## Methods

### Study design

This study employed a triangulated mixed-methods cross-sectional design to examine *human–GenAI interaction patterns* and their implications for educational outcomes and ethical tensions in nursing education. The study was grounded in the premise that the effects of generative AI are mediated not only by its availability, but by *how students interact with it* during learning processes.

The study was conducted in three integrated phases. First, rapid review mapped evidence on generative AI in nursing education, focusing on risks, benefits, and interaction gaps, following the PRISMA-ScR checklist and rapid review methods. Second, a quantitative survey examined students’ awareness, interaction tendencies, and usage intentions with DeepSeek, identifying patterns such as reliance, verification, and task-oriented use. Third, structured interviews explored students’ lived experiences, focusing on how they interpret AI outputs, integrate them into learning, and navigate ethical tensions. These strands were triangulated to provide a comprehensive analysis of human–GenAI interaction across cognitive, behavioral, and affective dimensions.

### Research questions

This study was guided by the following research questions:

1. What evidence exists regarding human interaction with generative AI (DeepSeek) in nursing education, and what gaps remain in understanding these interactions?
2. What are the dominant patterns of student interaction with DeepSeek in terms of awareness, engagement, and usage tendencies?
3. How do nursing students experience and interpret their interactions with DeepSeek, and what interaction-related ethical tensions emerge?
4. How do different interaction patterns influence educational outcomes, including cognitive, behavioral, and affective dimensions?

### Setting

The study was conducted at Youjiang Medical University for Nationalities in Guangxi, China, a leading institution in medical and nursing education recognized for integrating advanced educational technologies into its curriculum. The university provides a context where generative AI tools are increasingly embedded in learning processes, making it particularly suitable for examining *human–GenAI interaction in authentic educational settings*.

### Participants

The study targeted 1,946 nursing graduate students. Using simple random sampling with proportionate allocation, a minimum sample of 321 was determined via Raosoft (95% confidence level; 5% margin of error). To address non-response, the sample was increased by 25%, yielding 401 participants. For the qualitative phase, purposive sampling selected 30 students for structured interviews, with sample size guided by data saturation to capture diverse interaction patterns and ethical tensions.

### Data collection

Current evidence regarding the use of generative artificial intelligence in nursing education was identified and synthesized, with a specific focus on human–GenAI interaction, educational outcomes, and ethical implications. The review was conducted using the methodological recommendations for rapid reviews, as well as the Preferred Reporting Items for Systematic Reviews and Meta-Analyses Extension for Scoping Reviews (PRISMA-ScR) guidelines.

Literature search was carried out using databases such as PubMed/MEDLINE, Scopus, Web of Science, ScienceDirect, and SpringerLink. The search was conducted in a manner that focused on the most up-to-date research on generative AI, restricting the time range of studies from January 2023 to March 2026, with seminal methodological and conceptual papers included where they were pertinent to provide context to the studies. The reference lists of articles that met the criteria were also hand searched for additional articles of relevance.

A combination of both controlled vocabulary and free-text keywords in a search strategy was used and logical operators (AND, OR, NOT) were used. Search strings entered by the representatives were: (“Artificial Intelligence” OR “Generative Artificial Intelligence” OR “Large Language Model” OR “ChatGPT” OR “DeepSeek”) AND (“Nursing Education” OR “Nursing Students” OR “Healthcare Education”) AND (“Human-AI Interaction” OR “Student Engagement” OR “Ethics” OR “Academic Integrity” OR “Critical Thinking”). Each term was reworded using the indexing system and search interface of each database, with the aim of maximizing the retrieval of literature relevant to the topic.

4,117 records were found in the first database search. Citations after duplicate removal and title and abstract screening were selected for full-text evaluation for those studies that focused on the use of artificial intelligence in nursing education. After screening for eligibility according to the pre-stated inclusion and exclusion criteria, 95 studies were selected for the rapid review and nine of these studies specifically investigated DeepSeek or DeepSeek in nursing education and related healthcare educational environments. Screening and selection of studies followed a consistent screening process, with the first researcher doing the main screening while about 20% of the screened articles were independently checked by a second researcher for consistency and to reduce selection bias, again following the rapid review methodology. The review aimed to provide an overview of the current state of research, outline gaps in knowledge, and define the rationale for this study on human–GenAI interaction, rather than conduct a comprehensive systematic review.

### Data analysis

Consistent with rapid review methodology, screening was conducted by one researcher with a 20% verification by a second reviewer. A formal methodological quality appraisal or risk-of-bias assessment of the included studies was not conducted because the objective of the rapid review was to map the current evidence, identify knowledge gaps, and inform the empirical component of the study rather than to synthesize intervention effectiveness or generate evidence-based recommendations. This approach is consistent with the purpose of rapid evidence mapping and scoping-oriented reviews.

Quantitative data were analyzed using SPSS version 29. Descriptive statistics were used to summarize awareness, interaction tendencies, and usage intention. Correlation analysis examined relationships among these variables, while multiple regression analysis identified predictors of usage intention and interaction behaviors.

Interview transcripts were analyzed using thematic analysis. Coding focused on identifying recurring interaction patterns, including how students engage with, interpret, and act upon AI outputs. Themes were iteratively refined to capture both functional uses of AI and associated ethical tensions. Findings from the rapid review, survey, and qualitative analysis were integrated through a process of triangulation. This allowed for the identification of convergent and divergent patterns, linking interaction types with observed educational outcomes and ethical implications.

**Table 1:** Summary of rapid review articles on DeepSeek in nursing education

Author (year)	Title	Country	Aim/focus
Allen, [2]	*DeepSeek and AI innovation: How Chinese universities broke through the glass ceiling of technological advancement*	China/USA	Discusses AI innovation (including DeepSeek) in higher education, with relevance to nursing education.
Cheng, [10]	*Application prospects of DeepSeek in nursing theoretical teaching*	China	Explores the potential applications of DeepSeek in nursing theory teaching.
Desmedt, [18]	*Trialing DeepSeek*,* the new kid on the artificial intelligence block*	Europe	Examines the use of DeepSeek in clinical nursing, relevant to nursing education and practice.
Gurbuz, [26]	*Comparative Efficacy of ChatGPT and DeepSeek in Addressing Patient Queries on Gonarthrosis and Total Knee Arthroplasty*	Turkey	Compares DeepSeek and ChatGPT for clinical queries, with relevance for nursing education in clinical settings.
Lv, [33]	*DeepSeek-powered locally deployed closed-loop system for electronic nursing documentation quality control*	China	Presents DeepSeek’s application in nursing documentation quality control, an important aspect of nursing education and practice.
Temsah et al. [48]	*DeepSeek in healthcare: Revealing opportunities and steering challenges of a new open-source artificial intelligence frontier*	Saudi Arabia	Reviews DeepSeek’s opportunities and challenges in healthcare, including implications for nursing education.
Wang, Y.-M., & Chen, T.-J. [50]	*The rise of AI in healthcare education: DeepSeek and GPT-4o take on the 2024 Taiwan pharmacist exam*	Taiwan	Compares DeepSeek and GPT-4o in healthcare education, relevant to nursing education.
Yan et al. [51]	*Comparative analysis of DeepSeek-V3*,* DeepSeek-R1*,* and OpenAI models in urology practice*	China	Analyzes DeepSeek models in urology; findings may inform nursing education in specialized fields.
Yao et al. [52]	*Chinese generative AI models (DeepSeek and Qwen) rival ChatGPT-4 in ophthalmology queries with excellent performance in Arabic and English*	China	Compares DeepSeek and other AI models in clinical queries, applicable to nursing education in medical domains.

## Results and discussion

### Evidence on human–GenAI interaction in nursing education and remaining gaps

The rapid review addressed the first research question by examining the current state of evidence on human interaction with generative AI, particularly DeepSeek, in nursing education. From the 4,117 records initially identified through database searching, 95 studies (2.31%) met the eligibility criteria for AI in nursing education, whereas only 9 studies (9.47% of eligible studies; 0.22% of all retrieved records) specifically examined DeepSeek in nursing education or closely related healthcare learning contexts. As shown in Table 1, these studies were concentrated in three main areas: conceptual or review-based discussions of DeepSeek in higher education and healthcare, exploratory applications in nursing teaching and documentation, and comparative evaluations of DeepSeek against other large language models in clinical or educational tasks.

The existing evidence remains limited and heavily weighted toward technical performance, application prospects, and broad conceptual discussions. This emphasis likely reflects the recent emergence of DeepSeek, where research has prioritized evaluating model capabilities before investigating learner behaviors in authentic educational settings. As a result, current studies describe what DeepSeek can do, but provide limited insight into how students interact with it during learning.

Studies by Cheng [10] and Lv et al. [33] suggest that DeepSeek may support nursing theory instruction and documentation processes, while comparative studies by Gurbuz [26], Wang and Chen [50], Yan et al. [51], and Yao et al. [52] show that the model performs competitively in selected educational and clinical tasks. Likewise, review-oriented works such as Allen [2], Desmedt [18], and Temsah et al. [48] place DeepSeek within the broader AI transformation in higher education and healthcare. However, this body of evidence remains largely technology centered. It describes what DeepSeek can do, but not how students actually interact with it during learning.

A central gap emerging from the review is the absence of interaction-level analysis. None of the identified studies systematically examined how learners formulate prompts, interpret outputs, verify information, or incorporate AI-generated responses into academic decision-making. This gap is consequential because many of the concerns emphasized in the broader literature on AI in nursing education—such as misinformation, overreliance, dishonesty, and weakened critical judgment—are not simply features of the tool itself, but consequences of how users engage with it [16, 24, 43, 53]. The rapid review therefore supports the need for the present study by showing that empirical research on human–GenAI interaction patterns in nursing education remains underdeveloped despite the growing interest in DeepSeek.

### Dominant patterns of student interaction with DeepSeek in terms of awareness, engagement, and usage tendencies

#### Descriptive statistics and factor structure of interaction patterns

To address the second research question, descriptive statistics and exploratory factor analysis were conducted to identify the dominant patterns of interaction with DeepSeek. As presented in Table 2, students reported moderately high awareness of DeepSeek (M = 4.02, SD = 0.81) and high usage intention (M = 4.12, SD = 0.78). Four interaction dimensions emerged from the factor analysis: passive interaction, efficiency-driven interaction, dependency-based interaction, and critical engagement. Together, these factors explained 68.4% of the total variance, indicating a stable interaction structure.

As shown in Table 2, the most dominant interaction pattern was efficiency-driven interaction, suggesting that students primarily used DeepSeek to accelerate academic tasks, simplify complex concepts, and improve productivity. This finding appears consistent with previous work highlighting generative AI as a tool for efficiency, immediate feedback, and learning support [9, 13, 21]. At the same time, the prominence of this interaction pattern suggests that students may approach AI instrumentally, valuing speed and convenience over reflective engagement. Such a pattern is pedagogically significant because efficiency gains may coexist with shallower processing, reduced metacognitive monitoring, and lower conceptual depth [30, 43]. One possible explanation is that immediate AI-generated answers reduce the need for students to independently analyze problems, compare alternative solutions, or reflect on their reasoning process, thereby limiting opportunities for deeper learning.

Dependency-based interaction also registered at a moderate level, indicating that students were not merely using AI occasionally but were increasingly incorporating it into routine learning behavior. This pattern suggests the externalization of cognitive work to AI systems, echoing concerns that generative AI may weaken self-regulated learning and independent problem-solving when used as a substitute rather than a scaffold [5, 19, 38]. As students become accustomed to relying on AI for routine academic tasks, they may gradually transfer cognitive responsibilities to the technology, reducing opportunities to strengthen independent problem-solving and clinical reasoning skills that are essential in nursing practice. By contrast, critical engagement had the lowest mean among the four patterns. This implies that although students demonstrated awareness of AI, fewer were consistently verifying outputs, cross-checking responses, or using AI in a deliberately reflective way. The difference between awareness and critical engagement suggests that knowledge about AI risks does not automatically translate into responsible interaction practices, reinforcing arguments for stronger AI literacy and pedagogical scaffolding [6, 17, 28]. This may occur because recognizing potential risks is insufficient without structured learning experiences that develop students’ skills in verifying information, evaluating evidence, and critically interpreting AI-generated outputs.

**Table 2:** Descriptive statistics and factor structure of interaction patterns

Construct	Mean	SD	Factor Loading Range	Interpretation
Awareness	4.02	0.81	—	Moderately high
Passive Interaction	3.85	0.88	0.71–0.84	Moderate
Efficiency Interaction	4.12	0.78	0.69–0.86	High
Dependency Interaction	3.92	0.89	0.73–0.88	Moderate
Critical Engagement	3.68	0.82	0.66–0.81	Moderate
Usage Intention	4.12	0.78	—	High

Note. Exploratory factor analysis identified four interaction dimensions. Total variance explained = 68.4%. Higher mean values indicate stronger endorsement of the construct

**Table 3:** Correlation matrix of awareness, interaction patterns, and usage intention

Variable	1	2	3	4	5
1. Awareness	—				
2. Efficiency Interaction	0.49***	—			
3. Dependency Interaction	-0.18**	0.42***	—		
4. Critical Engagement	0.36***	0.28***	-0.31***	—	
5. Usage Intention	0.51***	0.57***	0.44***	0.29***	—

Note. Pearson product-moment correlations were used. ***p < .001. **p < .01

**Table 4:** Multiple regression analysis predicting usage intention from awareness and interaction patterns

Predictor	β	SE	t	*p*	Interpretation
Awareness	0.38	0.04	9.42	<0.001***	Significant positive predictor
Efficiency Interaction	0.41	0.05	10.11	<0.001***	Strongest predictor
Dependency Interaction	0.22	0.04	5.89	<0.001***	Moderate positive predictor
Critical Engagement	0.12	0.03	2.94	0.003**	Weak but significant predictor

Model Fit: R² = 0.52, Adjusted R² = 0.51, F(4,396) = 107.63, *p* < .001

Note. Dependent variable = Usage intention. Standardized beta coefficients are reported. ***p < .001. **p < .01

#### Relationships between interaction patterns and usage intention

As indicated in Table 3, awareness was positively associated with efficiency interaction, critical engagement, and usage intention, suggesting that students with stronger understanding of DeepSeek were also more likely to use it frequently and strategically. The strongest relationship was observed between efficiency interaction and usage intention (*r* = 0.57, *p* < 0.001), representing a moderately strong positive correlation. In contrast, dependency interaction was negatively correlated with critical engagement (*r* = − 0.31, *p* < 0.001), indicating that students who relied more heavily on DeepSeek tended to report lower levels of verification and reflective evaluation of AI-generated information. This is consistent with literature showing that perceived utility is a major driver of AI adoption in educational settings [27, 28].

At the same time, the negative correlation between dependency interaction and critical engagement (*r* = –0.31, *p* < 0.001) is especially important. This pattern suggests that greater reliance on AI is associated with lower levels of verification, reflection, and analytical scrutiny. In other words, as students become more dependent on DeepSeek, they appear less likely to challenge, contextualize, or independently validate its outputs. This finding reinforces concerns that unchecked AI use can encourage passive learning and cognitive offloading, particularly in environments where rapid completion of tasks is rewarded more strongly than reflective practice [15, 16, 37]. A plausible explanation is that repeated reliance on AI-generated responses can increase users’ confidence in the technology, reducing the perceived need to verify information through textbooks, clinical guidelines, or instructor feedback. The results also suggest that efficiency and dependency may coexist students may find AI useful and convenient while simultaneously becoming more reliant on it in ways that reduce deeper engagement.

#### Predictors of usage intention

As shown in Table 4, the regression model explained 52% of the variance in usage intention (R² = 0.52), indicating substantial explanatory power. Efficiency interaction emerged as the strongest predictor, followed by awareness, while dependency interaction and critical engagement also remained statistically significant predictors of students’ intention to continue using DeepSeek. This suggests that students’ intention to continue using DeepSeek is shaped primarily by two factors: their recognition of its utility and their broader familiarity with AI. This pattern is consistent with prior studies showing that both functional usefulness and AI literacy drive acceptance and integration of generative AI tools [17, 23]. Students who perceive AI as useful for improving learning efficiency are naturally more inclined to incorporate it into their routine academic activities, particularly when it reduces workload and provides immediate access to information.

Notably, dependency interaction also exerted a significant positive effect on usage intention. Although this may seem beneficial at first glance, it is pedagogically ambivalent. A positive relationship between dependency and intention implies that students may become more committed to continued AI use precisely when they have begun to rely on it cognitively. This supports concerns in the literature that growing dependence on AI can normalize convenience-based learning while eroding independent reasoning and self-regulatory habits [5, 19, 38]. Critical engagement was also a significant predictor, though its effect was smaller. This suggests that students who verify and interrogate AI outputs still value DeepSeek, but such use is more effortful and perhaps less immediately reinforcing than efficiency-driven use. Overall, the regression results indicate that DeepSeek use is sustained not only by informed and reflective engagement, but also by convenience and dependence, underscoring the need to distinguish beneficial from potentially harmful interaction patterns.

### How nursing students experience and interpret their interactions with deepseek, and the ethical tensions that emerge

The qualitative findings deepened the quantitative results by showing how students interpreted their interaction with DeepSeek in everyday academic situations. Four recurrent patterns were identified—passive consumption, efficiency-driven interaction, dependency-based interaction, and critical engagement—alongside cross-cutting ethical tensions involving accuracy, integrity, trust, and professional responsibility.

Students who describe passive interaction often equate fluency with reliability. Their accounts suggested that well-phrased AI outputs were sometimes accepted without sufficient scrutiny, especially under pressure. One participant stated, *“Sometimes I just accept what DeepSeek gives because it sounds very professional and complete. I feel like it already explains everything*,* so I don’t question it anymore. Only later I realize some parts are incorrect or too general.”* Another participant shared, *“There are times when I rely on it too quickly*,* especially when I am tired or under pressure. I don’t always check if the information matches my textbooks*,* which makes me unsure if I am learning correctly.”* These narratives indicate that interaction with AI is shaped not only by capability but also by cognitive fatigue, academic pressure, and the persuasive fluency of generated text. This reflects broader concerns that large language models can encourage acceptance-based learning by presenting inaccurate information in a confident and coherent form [47, 49].

Accounts of efficiency-driven interaction were often positive, particularly in relation to exam preparation, simplification of difficult topics, and timesaving. One participant explained, *“DeepSeek helps me understand difficult topics faster*,* especially when I don’t have time to read everything. But sometimes I feel like I only understand the surface and not the full concept.”* Another said, *“It gives me quick answers*,* which is very helpful*,* but I notice I don’t spend as much time thinking through problems anymore.”* These responses show that students recognize both the benefits and the limitations of efficiency-based AI use. While DeepSeek improves convenience and confidence, it may also compress the time normally devoted to reflection and reasoning. This is consistent with prior work suggesting that AI can enhance educational efficiency while also encouraging shortcut-oriented engagement if left pedagogically unstructured [13, 21, 30].

The theme of dependency emerged strongly in students’ narratives about changing study habits. One participant reflected, “I noticed that I depend on DeepSeek more than before. Even for simple questions, I go to it instead of trying to figure things out myself. It makes studying easier, but I feel less confident without it.” Another added, “When I don’t have access to it, I feel unprepared because I got used to using it for everything. It changed how I study.” These statements suggest that AI use can gradually reshape learning behavior, not just by assisting students but by becoming embedded in how they initiate, manage, and complete academic tasks. The concern here is not only frequency of use, but the displacement of independent cognitive effort. This supports literature arguing that generative AI can foster dependence and reduce learner autonomy if not framed as a support tool rather than a substitute for active learning [19, 37, 38].

A smaller but important group of participants described critical engagement, demonstrating more deliberate and evaluative use of DeepSeek. One student said, *“I use DeepSeek as a starting point*,* but I always check with textbooks or lecture notes. I don’t fully trust it because I know it can make mistakes.”* Another explained, *“It is helpful*,* but I treat it as a guide*,* not the final answer. I think that is important*,* especially in nursing where accuracy matters.”* These accounts illustrate a more mature form of human–AI interaction in which AI is positioned as a provisional aid rather than an unquestioned authority. Such students appeared to balance convenience with professional caution, aligning with emerging frameworks on AI literacy that stress verification, contextual judgment, and responsible interpretation [6, 28].

Across all four interaction patterns, three ethical tensions became especially visible. The first involved efficiency versus academic integrity, as students reported that the ease of obtaining polished outputs could normalize shortcut learning and reduce originality, echoing concerns raised by Alsadhan et al. [3] and Mohammad et al. [35]. The second involved trust versus reliability, as students recognized that DeepSeek could produce false references, incomplete explanations, or answers that contradicted formal sources, consistent with concerns about hallucination, fabricated citations, and misinformation [43, 46]. The third involved convenience versus professional responsibility, particularly in a discipline such as nursing where inaccurate knowledge may carry implications beyond the classroom. Students’ concern that AI-assisted learning could weaken clinical judgment resonates with the warnings of O’Connor [41] and Shin et al. [45], who argue that uncritical reliance on AI may undermine the formation of sound professional reasoning. Taken together, the qualitative findings show that the educational significance of DeepSeek lies not only in its functions, but in how students’ position, interpret, and trust it within their learning process.

### Interaction patterns influence educational outcomes across cognitive, behavioral, and affective dimensions

To examine how interaction patterns influenced educational outcomes, separate multiple regression analyses were conducted for critical thinking, academic motivation, and academic integrity risk. The results are shown in Table 5.

**Table 5:** Multiple regression analyses predicting educational outcomes from interaction patterns

Outcome Variable	Predictor	β	SE	t	*p*	95% CI	Interpretation
Critical Thinking	Passive Interaction	-0.34	0.05	-6.80	<0.001***	[-0.44, -0.24]	Significant negative predictor
	Efficiency Interaction	-0.12	0.04	-2.90	0.004**	[-0.20, -0.04]	Small negative effect
	Dependency Interaction	-0.29	0.05	-5.72	<0.001***	[-0.39, -0.19]	Moderate negative effect
	Critical Engagement	0.46	0.04	10.90	<0.001***	[0.38, 0.54]	Strong positive predictor
	Model Fit			F (4,396) = 91.52	<0.001***		R² = 0.48
Academic Motivation	Passive Interaction	-0.21	0.05	-4.22	<0.001***	[-0.31, -0.11]	Moderate decline
	Efficiency Interaction	0.18	0.04	4.12	<0.001***	[0.09, 0.27]	Positive influence
	Dependency Interaction	-0.33	0.05	-6.45	<0.001***	[-0.43, -0.23]	Strong negative effect
	Critical Engagement	0.29	0.04	6.87	<0.001***	[0.21, 0.37]	Strong positive effect
	Model Fit			F (4,396) = 71.33	<0.001***		R² = 0.42
Academic Integrity Risk	Passive Interaction	0.41	0.05	8.20	<0.001***	[0.31, 0.51]	Strong risk predictor
	Efficiency Interaction	0.22	0.04	5.01	<0.001***	[0.13, 0.31]	Moderate risk
	Dependency Interaction	0.37	0.05	7.25	<0.001***	[0.27, 0.47]	Strong risk predictor
	Critical Engagement	-0.28	0.04	-6.12	<0.001***	[-0.37, -0.19]	Protective factor
	Model Fit			F (4,396) = 103.22	<0.001***		R² = 0.51

Note. Separate multiple regressions were performed for each outcome variable. Standardized beta coefficients are reported. ***p < .001. **p < .01

As shown in Table 5, the effects of interaction patterns on educational outcomes were substantial and differentiated. For critical thinking, passive, efficiency-driven, and dependency-based interaction all showed negative effects, whereas critical engagement was a strong positive predictor. This indicates that merely using DeepSeek does not guarantee cognitive benefit; rather, the educational effect depends on how the tool is used. Efficiency-driven interaction may help students’ complete tasks quickly, but its negative association with critical thinking suggests that convenience can come at the expense of analytical depth. Dependency-based interaction showed an even stronger negative effect, indicating that students who increasingly outsource thinking to AI may undermine the very reasoning processes nursing education seeks to cultivate. In contrast, critical engagement appears to function as a cognitively protective and enhancing pattern, supporting reflection, verification, and deeper understanding. This is consistent with frameworks emphasizing active processing, reflective evaluation, and AI literacy as central to meaningful learning with generative AI [6, 28].

For academic motivation, the pattern was similarly differentiated. Efficiency interaction positively predicted motivation, suggesting that students may feel encouraged when AI makes learning more manageable and accessible. However, passive and dependency-based interaction reduced motivation, particularly dependency, which had the strongest negative effect. This suggests that when students over-rely on AI, the short-term ease it provides may gradually displace intrinsic motivation and self-directed effort. Such a pattern supports concerns that AI may weaken study discipline and learner autonomy if it becomes a substitute for active engagement [5, 19]. Critical engagement again showed a positive effect, suggesting that students who actively manage their use of DeepSeek are better able to sustain productive motivation without surrendering ownership of the learning process.

The model for academic integrity risk further clarifies the ethical dimension of these interaction patterns. Passive and dependency-based interaction were strong positive predictors of integrity risk, while efficiency interaction also showed a moderate positive effect. These findings suggest that the convenience and fluency of AI outputs may increase the likelihood of shortcut learning, overreliance, or misuse in academic tasks. By contrast, critical engagement functioned as a protective factor, reducing integrity risk. This indicates that the most educationally beneficial interaction pattern is also the most ethically protective one. These results align with the broader literature warning that generative AI may simultaneously support learning and intensify risks related to plagiarism, dishonesty, and uncritical acceptance unless students are guided toward reflective use [3, 16, 48].

**Table 6:** One-way ANOVA comparing educational outcomes across interaction profiles

Outcome	Interaction Group	Mean	SD	F	*p*	Post Hoc (Tukey)	Interpretation
Critical Thinking	Passive	2.98	0.85	38.12	<0.001***	Passive < Efficiency, Critical; Dependency < Critical; Efficiency < Critical	Significant group differences
	Efficiency	3.45	0.79				
	Dependency	3.12	0.88				
	Critical Engagement	4.12	0.74				Highest mean
Academic Motivation	Passive	3.20	0.83	22.45	<0.001***	Dependency < Efficiency, Critical; Passive < Critical	Significant group differences
	Efficiency	3.98	0.75				
	Dependency	3.01	0.88				Lowest mean
	Critical Engagement	4.05	0.71				Highest mean

Note. One-way ANOVA with Tukey post hoc comparisons was used to examine group differences across interaction profiles. ***p < .001

**Table 7:** Mediation analysis examining dependency as a mediator between efficiency interaction and critical thinking

Path	β	SE	*p*	Interpretation
Efficiency Interaction → Dependency Interaction	0.42	0.04	<0.001***	Strong positive relationship
Dependency Interaction → Critical Thinking	-0.36	0.05	<0.001***	Significant negative relationship
Indirect Effect of Efficiency on Critical Thinking via Dependency	-0.15	0.03	<0.001***	Significant mediation effect

Note. Mediation analysis was conducted using PROCESS Model 4 with bootstrapped standard errors. ***p < .001

**Fig. 1:**
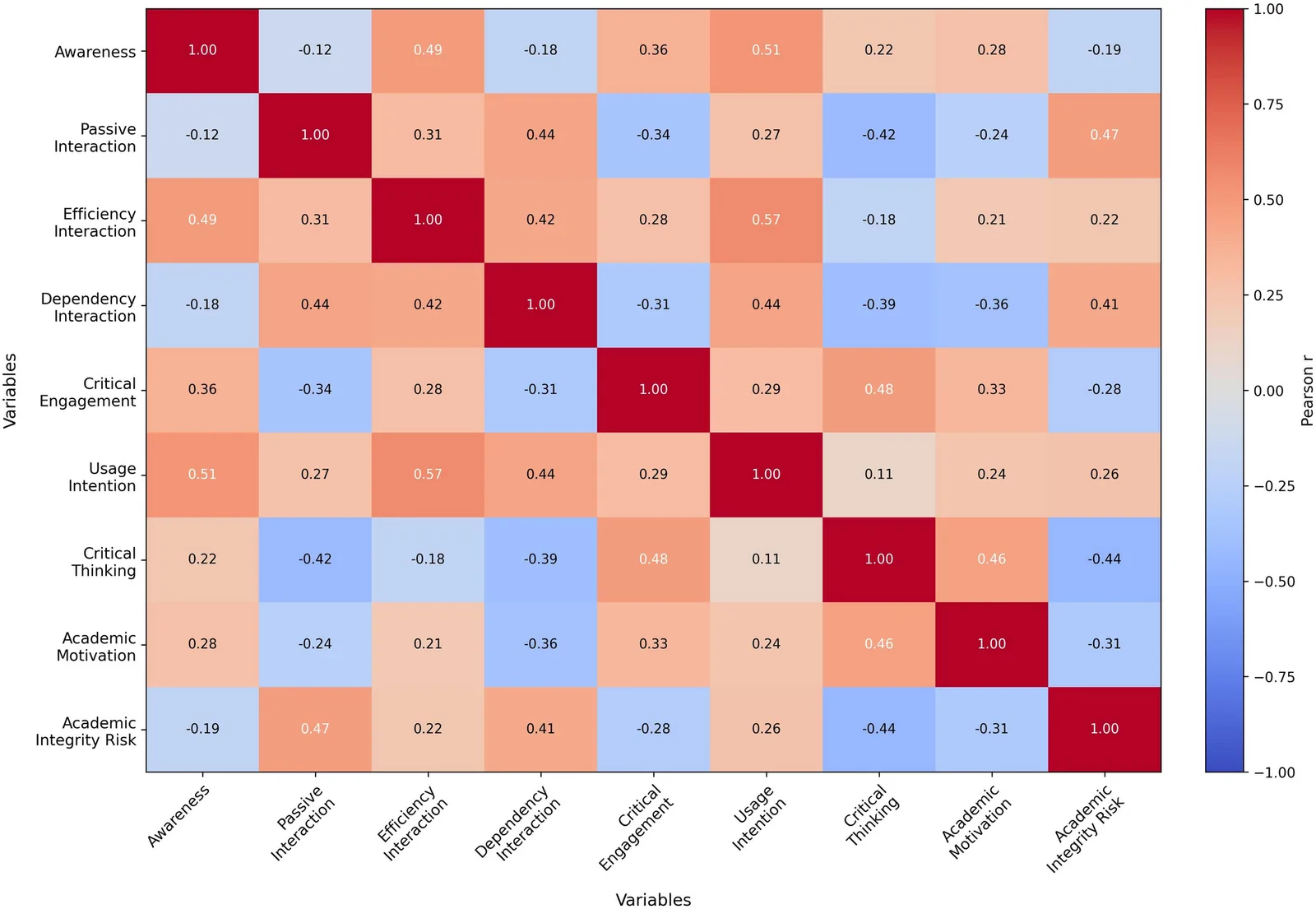
Correlation heatmap of awareness, interaction patterns, and educational outcomes. A correlation heatmap showing relationships among awareness, interaction patterns, usage intention, and educational outcomes (*n* = 401). Efficiency interaction is strongly associated with usage intention (*r* = .57), while dependency also shows a positive relationship (*r* = .44) but is negatively linked to critical engagement (*r* = –.31). Passive and dependency interactions are negatively correlated with critical thinking (*r* = –.42, *r* = –.39) and positively associated with academic integrity risk (*r* = .47, *r* = .41). In contrast, critical engagement is positively related to critical thinking (*r* = .48) and motivation (*r* = .33), and negatively associated with integrity risk (*r* = –.28)

To complement the regression findings, one-way ANOVA was conducted to compare educational outcomes across the four dominant interaction profiles. As indicated in Table 6, students classified under the critical engagement profile obtained the highest mean scores for both critical thinking and academic motivation, whereas those in the passive and dependency profiles performed less favorably. These group differences reinforce the regression findings by showing that interaction patterns are associated not only with variable-level trends but with clearly distinguishable educational profiles. The critical engagement group appears to represent the most adaptive pattern of human–AI interaction, balancing AI utility with independent judgment. By contrast, the passive and dependency groups appear more vulnerable to reduced cognitive engagement.

As shown in Fig. 1, the correlation heatmap visually summarizes the relationships among awareness, interaction patterns, usage intention, and educational outcomes. The figure shows that efficiency interaction is strongly associated with usage intention, whereas critical engagement is linked to better educational outcomes and lower academic integrity risk. In contrast, passive and dependency-based interactions are associated with poorer cognitive and ethical outcomes.

These differences highlight the trade-offs embedded in AI-mediated learning. Efficiency can improve productivity and perceived support, but when it is not accompanied by evaluation and verification, it risks sliding into passivity or dependency. This supports the argument that educational outcomes are not determined by the presence of AI itself, but by the interaction logic through which students engage it. In nursing education, where judgment, accuracy, and accountability are central, this distinction is especially important. The findings therefore extend existing concerns about the dual-edged nature of generative AI by showing that its benefits and risks are mediated by identifiable interaction profiles rather than by technology use alone [24, 48, 53].

To further clarify the mechanism linking interaction patterns to outcomes, a mediation analysis was conducted using PROCESS Model 4. As shown in Table 7, dependency significantly mediated the relationship between efficiency interaction and critical thinking. This suggests that efficiency-oriented use of DeepSeek does not necessarily reduce critical thinking directly; rather, it may do so indirectly by fostering greater reliance on AI. In practical terms, students may begin by using DeepSeek for convenience and time-saving, but repeated efficiency-based use can gradually evolve into dependency, which in turn weakens critical reasoning. This finding provides preliminary evidence suggesting that the educational cost of AI may unfold through interaction trajectories rather than through immediate effects alone. In other words, efficiency-oriented use may initially appear beneficial, but repeated reliance on AI can gradually evolve into dependency, which subsequently reduces opportunities for students to practice independent reasoning and critical evaluation. It also helps explain why generative AI can appear beneficial in the short term while producing problematic learning consequences over time. Such a mechanism is consistent with arguments that AI can facilitate convenience and performance while simultaneously undermining independent thought if not accompanied by reflective safeguards [27, 30, 41].

## Conclusions

The study reveals that the integration of generative AI in nursing education is defined by a set of critical ethical tensions and educational trade-offs that are embedded within how students engage with the technology. Three central tensions emerge. First, efficiency versus academic integrity: while DeepSeek enables rapid task completion and improved productivity, it simultaneously facilitates shortcut learning, normalization of AI-assisted dishonesty, and reduced originality in student work. Second, trust versus reliability: students frequently rely on AI outputs that appear authoritative but may contain inaccuracies, fabricated references, or content misaligned with clinical standards, creating a dangerous gap between perceived and actual knowledge. Third, convenience versus professional responsibility: the ease of accessing AI-generated answers reduces effortful learning and critical reasoning, raising concerns about preparedness for real-world clinical decision-making where accuracy and judgment are essential. These tensions demonstrate that ethical risks are not peripheral issues but are directly produced through everyday interaction practices with AI.

Closely tied to these tensions are clear educational trade-offs. Gains in efficiency and accessibility often come at the expense of critical thinking, self-regulated learning, and intrinsic motivation, particularly under passive and dependency-based interaction patterns. While students benefit from faster comprehension and increased confidence, these advantages are frequently offset by superficial understanding and reduced analytical engagement. In contrast, critical engagement emerges as the only pattern that balances benefits and risks, supporting deeper learning while mitigating ethical concerns—yet it remains the least practiced. The study’s key practical contribution is therefore the identification of interaction quality—not AI use itself—as the decisive factor in educational outcomes. This underscores the urgent need for higher education, particularly nursing programs, to move beyond permissive or restrictive AI policies and instead implement structured, interaction-focused guidance that cultivates verification, reflection, and disciplined use of AI, ensuring that technological efficiency does not undermine the cognitive, ethical, and professional foundations of nursing education.

## Supplementary Information

Below is the link to the electronic supplementary material.


Supplementary Material 1


## Data Availability

The datasets generated and/or analyzed during the current study are not publicly available due to ethical considerations and participant confidentiality but are available from the corresponding author on reasonable request.
