# Supplementary material for: From adoption to interaction: examining human-GenAI engagement patterns, ethical tensions, and educational trade-offs in nursing education
Source: BMC Nurs. 2026 Jul 31;25:787. doi: 10.1186/s12912-026-05132-7 (PMC13536568; doi:10.1186/s12912-026-05132-7)
Supplement: Supplementary file 1 [file 12912_2026_5132_MOESM1_ESM.docx]

**Focus Interview Guide**

**I. Introduction and Awareness of DeepSeek**

1. Before participating in this study, what did you know about DeepSeek and its role in nursing education?
2. How did you first learn about DeepSeek, and what were your initial thoughts on its potential benefits and risks?

**II. Experiences Using DeepSeek**

3. Can you describe a situation where you relied on DeepSeek in your coursework? What was the outcome?
4. How does using DeepSeek affect the amount of time, effort, and thinking you spend on completing coursework, studying, or academic tasks?
5. Have you ever received answers from DeepSeek that were wrong, inconsistent, or misleading? How did that affect your trust in it?
6. Do you feel that using DeepSeek has changed your study habits or reliance on textbooks and lectures? If so, how?

**III. Ethical Gaps and Risks**

7. In your experience, what ethical problems arise when students use DeepSeek (e.g., plagiarism, academic dishonesty, overreliance)?
8. Do you feel that using DeepSeek weakens critical thinking, originality, or decision-making skills? Why or why not?
9. Are you concerned about privacy or security risks when entering information into DeepSeek? Please explain.
10. Have you noticed situations where DeepSeek’s responses showed bias, fabricated references, or contradictions? How do you interpret these issues?
11. Do you think all students have equal access to DeepSeek? How might this create fairness or inequality problems in nursing education?

**IV. Reflections and Recommendations**

12. What do you believe is the biggest ethical risk of relying on DeepSeek in nursing education?
